# Prospective comparison of the diagnostic accuracy of 18F-FDG PET/MRI, MRI, CT, and bone scintigraphy for the detection of bone metastases in the initial staging of primary breast cancer patients

**DOI:** 10.1007/s00330-021-07956-0

**Published:** 2021-04-28

**Authors:** Nils Martin Bruckmann, Julian Kirchner, Lale Umutlu, Wolfgang Peter Fendler, Robert Seifert, Ken Herrmann, Ann-Kathrin Bittner, Oliver Hoffmann, Svjetlana Mohrmann, Christina Antke, Lars Schimmöller, Marc Ingenwerth, Katharina Breuckmann, Andreas Stang, Christian Buchbender, Gerald Antoch, Lino M. Sawicki

**Affiliations:** 1 Department of Diagnostic and Interventional Radiology, Medical Faculty, University Dusseldorf, Moorenstrasse 5, D-40225 Dusseldorf, Germany; 2 Department of Diagnostic and Interventional Radiology and Neuroradiology, University Hospital Essen, University of Duisburg-Essen, D-45147 Essen, Germany; 3 Department of Nuclear Medicine, University Hospital Essen, University of Duisburg-Essen, D-45147 Essen, Germany; 4 Department Gynecology and Obstetrics, University Hospital Essen, University of Duisburg-Essen, D-45147 Essen, Germany; 5 Department of Gynecology, Medical Faculty, University Dusseldorf, D-40225 Dusseldorf, Germany; 6 Department of Nuclear Medicine, Medical Faculty, University Dusseldorf, 40225 Dusseldorf, Germany; 7 Institute of Pathology, University Duisburg-Essen and the German Cancer Consortium (DKTK), University Hospital Essen, West German Cancer Center, Essen, Germany; 8 Institute of Medical Informatics, Biometry and Epidemiology, University Hospital of Essen, Essen, Germany

**Keywords:** Multimodal imaging, Positron emission tomography, Tomography, X-ray, Radionuclide imaging, Breast neoplasms

## Abstract

**Objectives:**

To compare the diagnostic performance of [^18^F]FDG PET/MRI, MRI, CT, and bone scintigraphy for the detection of bone metastases in the initial staging of primary breast cancer patients.

**Material and methods:**

A cohort of 154 therapy-naive patients with newly diagnosed, histopathologically proven breast cancer was enrolled in this study prospectively. All patients underwent a whole-body [^18^F]FDG PET/MRI, computed tomography (CT) scan, and a bone scintigraphy prior to therapy. All datasets were evaluated regarding the presence of bone metastases. McNemar *χ*^2^ test was performed to compare sensitivity and specificity between the modalities.

**Results:**

Forty-one bone metastases were present in 7/154 patients (4.5%). Both [^18^F]FDG PET/MRI and MRI alone were able to detect all of the patients with histopathologically proven bone metastases (sensitivity 100%; specificity 100%) and did not miss any of the 41 malignant lesions (sensitivity 100%). CT detected 5/7 patients (sensitivity 71.4%; specificity 98.6%) and 23/41 lesions (sensitivity 56.1%). Bone scintigraphy detected only 2/7 patients (sensitivity 28.6%) and 15/41 lesions (sensitivity 36.6%). Furthermore, CT and scintigraphy led to false-positive findings of bone metastases in 2 patients and in 1 patient, respectively. The sensitivity of PET/MRI and MRI alone was significantly better compared with CT (*p* < 0.01, difference 43.9%) and bone scintigraphy (*p* < 0.01, difference 63.4%).

**Conclusion:**

[^18^F]FDG PET/MRI and MRI are significantly better than CT or bone scintigraphy for the detection of bone metastases in patients with newly diagnosed breast cancer. Both CT and bone scintigraphy show a substantially limited sensitivity in detection of bone metastases.

**Key Points:**

• *[*^*18*^*F]FDG PET/MRI and MRI alone are significantly superior to CT and bone scintigraphy for the detection of bone metastases in patients with newly diagnosed breast cancer.*

• *Radiation-free whole-body MRI might serve as modality of choice in detection of bone metastases in breast cancer patients.*

## Introduction

Breast cancer is by far the most common solid neoplasm in women worldwide and with 15% the leading cause of tumor-related deaths in women every year [1]. Once the diagnosis is confirmed, the prognosis of disease depends largely on the stage of its spread and choice of an adequate therapy. Additionally to the assessment of the extent of the primary tumor in the breast and locoregional lymph node involvement, the detection of distant metastases is crucial, since this can result in an extension of the irradiation field or an adjustment of chemotherapy and eventually in a change to a palliative therapy concept [2]. Therefore, imaging-based whole-body staging plays a pivotal role in the primary diagnostics of breast cancer patients with a high risk for the presence of distant metastases.

Despite the advances in the treatment of breast cancer, up to 30% of patients still develop distant metastases over the course of the disease [3]. Herein, the skeleton is the most frequent site of distant metastases in breast cancer patients, accounting for 50–70% of all metastases [3–7]. The affection of the bone can cause various complications such as pain, pathological fractures, spinal cord compression, and hypercalcemia, which often have a major impact on patients’ morbidity and mortality [8–10]. Early detection can help to better control the disease, minimize complications, and, as a result, achieve a better quality of life [9].

As the initial staging has become increasingly important in recent years, the diagnostic algorithm was adapted and a thoraco-abdominal CT as well as bone scintigraphy was implemented [11]. If available, a PET/CT examination can also be used in primary staging, but has been rarely applied so far due to its low availability and higher costs. Therefore, bone scintigraphy in combination with CT are widely considered to be the gold standard for the detection of bone metastases, and are also recommended as the methods of choice in current guidelines [12–14]. However, previous studies have suggested that MRI provides advantages in the detection of bone lesions when compared to bone scan and might top CT as most beneficial whole-body staging examination [9, 15]. Accordingly, MRI has been discussed as an alternative staging tool for breast cancer patients and has already been added as a method of choice in patients with neurological symptoms and signs which suggest the possibility of spinal cord compression in the latest 2018 and 2020 European Society For Medical Oncology (ESMO) guidelines [12, 14], but is rarely used in everyday clinical routine [16].

The application of hybrid imaging techniques has proven to be of additional benefit in this context [17–20]. However, the impact of a [^18^F]FDG PET/MRI examination for the detection of bone metastases in primary breast cancer patients has been scarcely investigated so far [21–23], and to the best of our knowledge, there is only a small cohort study investigating its role in comparison to conventional imaging for the detection of bone metastases in the initial staging of breast cancer [23].

Thus, the purpose of this study was to investigate and compare the diagnostic value of [^18^F]FDG PET/MRI, MRI alone, CT, and bone scintigraphy for the detection of bone metastases in the initial staging of primary breast cancer patients.

## Material and methods

### Patients

**Fig. 1:**
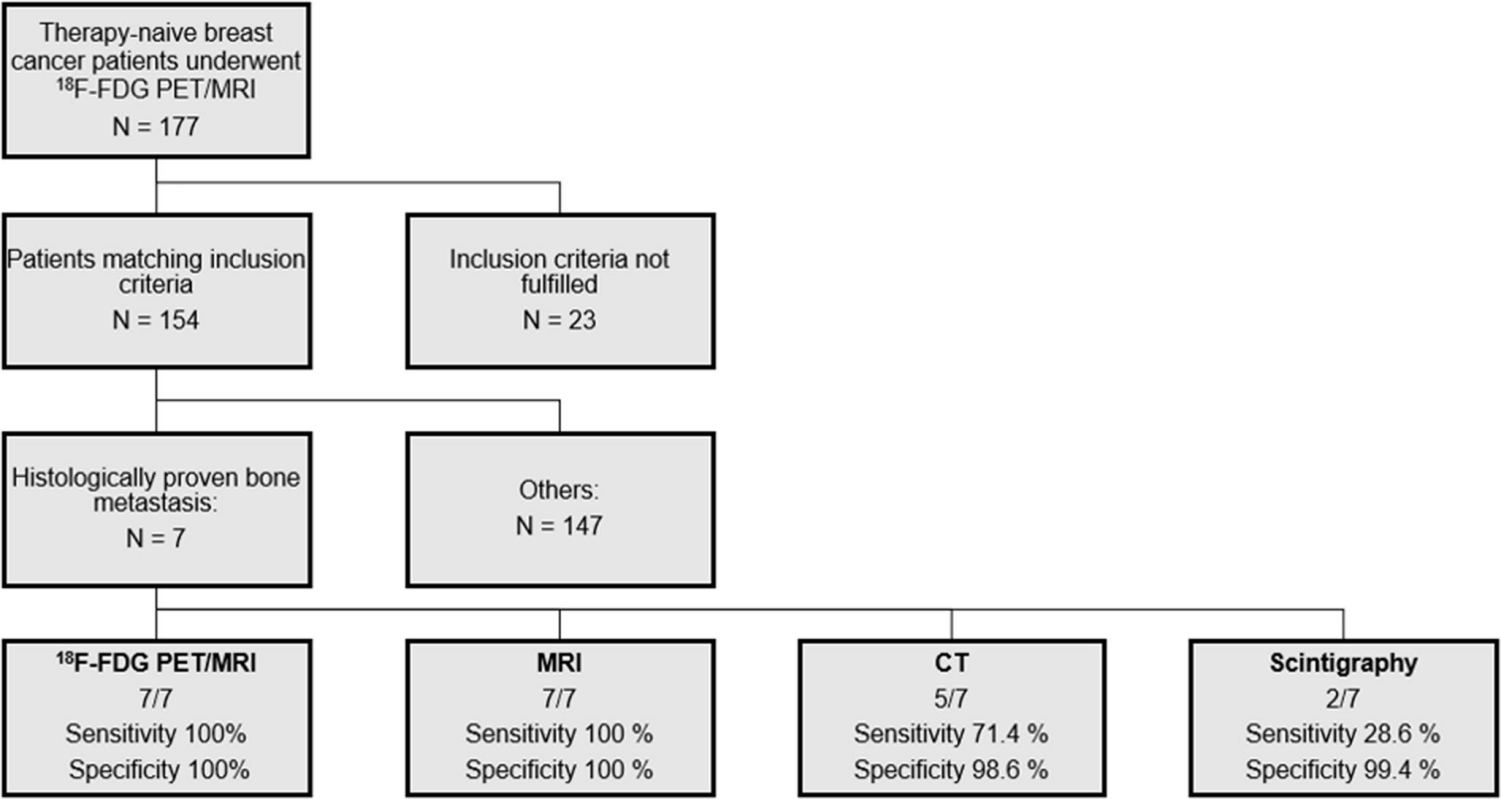
Flow-chart showing process of inclusion and patient-based specificity and sensitivity of each modality. of non-fulfilment of inclusion criteria are described in the text

This prospective study was approved by the institutional review board of the University of Duisburg-Essen (study number 17-7396-BO) and Düsseldorf (study number 6040R) and performed in conformance with the Declaration of Helsinki and its later amendments. Written informed consent form was obtained from all patients. The present study is a sub-analysis of a prospective, super-ordinate, main study (BU3075/2-1), and the research question of the present sub-analysis is markedly different from the main study. Inclusion criteria were defined as follows: (1) newly diagnosed, treatment-naive T2 tumor or higher T-stage, or (2) newly diagnosed, treatment-naive triple-negative tumor of every size, or (3) newly diagnosed, treatment-naive tumor with molecular high risk (T1c, Ki67 > 14%, HER2-new over-expression, G3). Exclusion criteria were contraindications to MRI or MRI contrast agents, missing imaging of a modality, pregnancy or breast-feeding, and former malignancies in the last 5 years. Inclusion criteria were chosen according to clinical ESMO guidelines to set elevated pre-test probability for distant metastases [12, 14]. Between March 2018 and March 2020, a total of 177 consecutive breast cancer patients underwent a [^18^F]FDG PET/MRI whole-body examination prior to therapy. Twenty-three patients had to be excluded from this study, because a comparable CT examination was missing in 7 patients and a bone scintigraphy in 17 patients, mainly due to patients not attending the examination appointment or the examination was performed in other medical institutions and were not available for evaluation. This resulted in a study cohort of 154 women (mean age 53.8 ± 11.9, range 30–82 years) (Fig. 1, Table 1).

**Table 1:** Histopathological data

		*N*
Total patients		154
Menopause status	Pre	63
	Peri	11
	Post	80
Family risk profile	Positive	42
	Negative	112
BRCA-1	Positive	4
	Negative	25
	Unknown	125
BRCA-2	Positive	2
	Negative	26
	Unknown	126
Ki 67	Positive (> 14%)	141
	Negative (< 14%)	13
PR status	Positive	107
	Negative	47
ER status	Positive	115
	Negative	39
HER2-neu expression	0	55
	1+	50
	2+	23
	3+	26
Subtype	Luminal A	7
	Luminal B	116
	HER2-enriched	3
	Basal-like	28
Tumor grade	G1	6
	G2	82
	G3	66
Histology	Ductal invasive/NST	136
	Lobular invasive	13
	Mucinous invasive	1
	Mixed type	1

### PET/MRI

All patients underwent a [^18^F]FDG PET/MRI examination on an integrated 3.0-Tesla Biograph mMR scanner (Siemens Healthineers) with a mean delay of 64 ± 17 min after [^18^F]FDG application. Prior to intravenous injection of a body weight–adapted dosage of [^18^F]FDG (4 MBq/kg body weight, mean activity: 254.4 ± 43.6 MBq), blood samples were obtained to ensure blood glucose levels below 150 mg/dl. All patients received a whole-body [^18^F]FDG PET/MRI from head to the mid-thigh in headfirst supine position.

PET images were obtained simultaneously with the MRI data with an acquisition time of 3 min per bed position in four or five positions (axial FOV: 25.8 cm; matrix size: 344 × 344; pixel size 2.09 × 2.09 mm). An iterative 3D ordinary Poisson ordered-subset expectation maximization (3D OP-OSEM) algorithm was conducted for reconstruction of PET images utilizing 3 iterations and 21 subsets, a Gaussian filter FWHM 4.0 mm, and a scatter correction. Depending on the patient’s height, up to 6-channel flex body coils, a dedicated 16-channel head-and-neck radiofrequency (RF) coil, and a 24-channel spine array RF coil were applied for MR imaging.

For tissue attenuation correction (AC) and scatter correction, a two-point (fat, water) transaxial acquired high-resolution CAIPIRINHA (CAIPI)-accelerated T1-weighted three-dimensional (3D) Dixon-VIBE (volume interpolated breath hold examination) sequence was acquired to generate a coronal four-compartment model attenuation map (umap, background air, lungs, fat, muscle). In addition, a bone atlas correction and a truncation correction as proposed by Blumhagen et al [24] was applied. Please see Table 2 for MRI protocol parameters. The PET/MRI scanner used was not time-of-flight (TOF) capable.

**Table 2:** Sequence parameters for the diagnostic MR-sequences and CT in staging of primary breast cancer patients

**CT**	**Region**	**Contrast agent**	**Orientation**	**mAs**	**kV**	**Speed (s per rotation)**	**Silence thickness (mm)**	**FOV (mm)**
Attenuation correction	Whole-body	No	Axial	80	120	0.75	4.0	600
Diagnostic CT	Thoraco-abdominal	Yes	Axial	210	120	0.75	4.0	350 × 459
**MR sequence**	**Region**	**Contrast agent**	**Orientation**	**TR (ms)**	**TE (ms)**	**Matrix size**	**Slice thickness (mm)**	**FOV (mm)**
EPI-DWI	Whole-body	No	Axial	11,900	86	192 × 144	5.0	380 × 285
T2w HASTE	Whole-body	No	Axial	1500	117	320 × 259	7.0	450 × 366
T1w fs VIBE	Whole-body	Yes	Axial	4.08	1.51	512 × 307	3.5	400 × 300

### Computed tomography

Two CT scanners (Definition Edge and Definition Flash, Siemens Healthineers) were used for thoraco-abdominal multi-slice contrast-enhanced CT using automated tube current modulation and tube voltage selection (CareDose 4D and CareKV, Siemens Heathineers). CTs were acquired in portal venous phase after intravenous application of a body weight–adapted dosage of non-ionic contrast agent (Table 2). The arms of the patients were placed upwards. Accordingly, only parts of the limbs that were pictured in the FOV of all modalities were included in the evaluation.

### Bone scintigraphy

Bone scintigraphy was performed according to a clinical routine protocol with planar whole-body scans using a dual-headed gamma camera equipped with low-energy high-resolution collimator (Symbia S, Siemens Healthineers). Three hours after intravenous injection of an average amount of 700 MBq of [^99m^Tc]-labeled polyphosphonate (HDP), anterior and posterior view scans were acquired with an acquisition time of 20 to 35 min. In all cases of uncertain radionuclide accumulations on bone scan, additional target images were taken or SPECT/CT images were acquired.

### Image analysis

In each patient, all examinations were performed over a 3-week period and prior to any oncologic therapy. The CT and PET/MRI datasets were analyzed separately and in random order by two radiologists experienced in hybrid and conventional imaging with a reading gap of 4 weeks to avoid recognition bias. Additionally, PET/MRI datasets and bone scintigraphy were also examined by a nuclear medicine physician. Discrepant findings were resolved by consensus decision-making in a separate session between the readers. For the evaluation of the MRI, images were separated from PET datasets. A picture archiving and communication system (Centricity; General Electric Medical Systems) and a dedicated image processing software OsiriX (Version 9.0.2, Pixmeo SARL) were used for image analysis. The readers were aware of the diagnosis but blinded to results of prior imaging.

The following criteria were applied to determine the presence of a bone metastasis in CT: a focal cortical destruction or increase of bone density, a focal bone expansion, periosteal reaction, pathological fractures, and contrast enhancement. In MRI signal intensity typical of metastasis in conventional MRI sequences, pathological contrast enhancement, diffusion restriction, pathological fractures, and bone edema were signs of malignancy. In [^18^F]FDG PET/MRI and on bone scan, a visually detectable focal uptake above background signal was considered a sign of malignancy. Besides lesion count, localization, and characterization (benign or malignant), the diagnostic confidence of every lesion in terms of its characterization as benign or malignant (5-point ordinal scale, 1 = very low confidence, 2 = low confidence, 3 = indeterminate confidence, 4 = high confidence, 5 = very high confidence) was assessed with each modality. The body volumes examined were chosen identically for each modality and covered the body from the thorax to mid-thighs.

### Reference standard

In all 154 women, diagnosis of primary breast cancer was confirmed histopathologically. Furthermore, in all patients with suspected osseous metastasis in any of the imaging modalities, at least one osseous lesion was histologically sampled. Due to clinical and ethical standards, a histological confirmation of some malignant lesions was not available, and a surrogate reference standard was applied taking into account all follow-up imaging. In all patients with suspected metastases, CT or MRI was performed as follow-up examination (mean delay 3.8 ± 1.3 month). In total, follow-up examinations were performed in 60 women, comprising 33 thoraco-abdominal CT, 22 whole-body MRI, and 5 patients receiving both examinations (mean delay 7.4 ± 5.1 month). The remaining patients, who did not undergo follow-up imaging, have been showing no clinical signs of bone metastases. Any increase of size or a decrease of size of suspicious lesions after therapy or newly occurred cortical destruction were regarded as signs of malignancy.

### Statistical analysis

Statistical analysis was performed using SPSS 24™ (IBM). Descriptive analysis was performed, and all data are presented as mean ± standard deviation including confidence intervals (CIs). To avoid statistical errors caused by clustered data (i.e., multiple observations within the same patient), all data were analyzed calculating sensitivity and specificity on a per-patient and a per-lesion basis. In addition, CIs have been adjusted using a ratio estimator, as described by Gender et al [25]. The lesion-based analysis was performed because knowledge of the exact number and localization of metastases can have large therapeutic impact, as solitary or oligometastases can be treated selectively with radiotherapy or surgery. For the comparison of sensitivity and specificity between the modalities, a McNemar *χ*^2^ test was performed. To assess the differences regarding the diagnostic confidence, a Wilcoxon signed rank test was applied. A *p* value < 0.05 was considered to indicate statistical significance.

## Results

### Patient-based analysis

According to the reference standard, bone metastases were present in 7/154 patients of the study cohort (4.5%). Both [^18^F]FDG PET/MRI and MRI alone were able to detect all of these patients. No false-positive patients were described by these two modalities. This resulted in a sensitivity of 100% (95% CI: 59.0–100.0) and a specificity of 100% (CI: 97.6–100.0). CT detected 5 of the 7 patients. In one patient with a single osteolytic metastasis, this was not visible on CT and in the other patient a small osteoblastic metastasis was misinterpreted as bone marrow island. Moreover, CT revealed false-positive findings in 2 non-metastasized patients, misinterpreting degenerative or posttraumatic lesions as malignant. This resulted in a sensitivity of 71.4% (CI: 35.9–91.8) and a specificity of 98.6% (CI: 95.2–99.6). Bone scintigraphy identified 2 of the 7 patients with bone metastases and showed a false-positive finding in a non-metastasized patient, resulting in a sensitivity of 28.6% (CI: 8.2–64.1) and a specificity of 99.4% (CI: 96.4–99.9). The McNemar *χ*^2^ test yielded a not significant difference in favor of [^18^F]FDG PET/MRI and MRI in comparison to CT in detecting true-positive patients (100% vs. 71.4%, *p* = 0.094) and in specificity (100% vs. 98.6%, *p* = 0.15) and a significant difference in sensitivity comparing [^18^F]FDG PET/MRI and MRI to bone scan (100% vs. 28.6%, *p* < 0.001). The difference between CT and bone scan yielded no statistical significance (71.4% vs. 28.6%, *p* = 0.076).

### Lesion-based analysis

A total of 45 bone lesions in 7 patients were included in the final evaluation, comprising 41 (91.1%) bone metastases and 4 (8.9%) benign bone lesions. One of the patients showed a diffuse infiltration of the entire axial skeleton, which was counted as 1 lesion. Twenty-three of the 41 metastases were classified as lytic, and 18 as sclerotic. Table 3 shows the localizations of all bone metastases. At least one lesion was confirmed by histopathological sampling in each patient; the remaining bone metastases were confirmed by follow-up imaging, according to the reference standard.

**Table 3:** Locations of all 41 bone metastases and number of detected lesions in each modality in comparison to the reference standard (in brackets). Most metastases affected the vertebrae and the pelvic bones. Mainly osteolytic metastases were missed/misinterpreted by CT and bone scan. Note that in CT arms were positioned upward and in PET/MRI besides the body. Only parts of the limbs that are pictured in the FOV of all modalities were evaluated

	PET/MRI and MRI		CT		Bone scan		Reference standard		
	Lytic	Sclerotic	Lytic	Sclerotic	Lytic	Sclerotic	Histology	CT	MRI
Vertebrae	9(9)	8(8)	4(9)	8(8)	1(9)	4(8)	1	12	4
Pelvic bones	6(6)	4(4)	2(6)	4(4)	3(6)	3(4)	5	3	2
Ribs	4(4)	1(1)	1(4)	0(1)	1(4)	0(1)	1	3	1
Limbs	4(4)	5(5)	1(4)	3(5)	2(4)	1(5)	1	5	3
Total	23(23)	18(18)	8(23)	15(18)	7(23)	8(18)	8	23	10

**Fig. 2:**
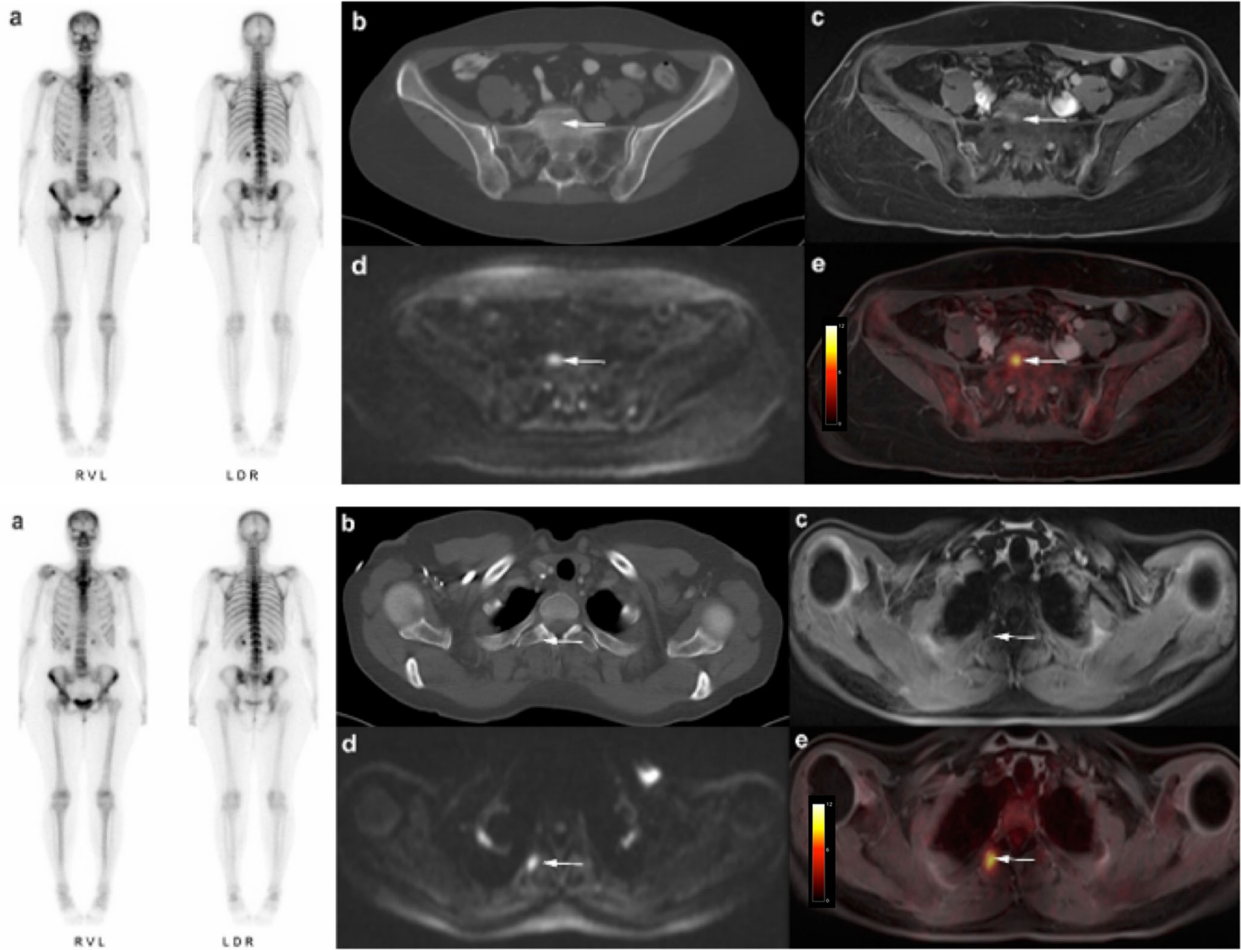
Fifty-eight-year-old woman with breast cancer and histologically proven bone metastases in the os sacrum and the second right rib. Clear evidence of metastatic infestation in fused [^18^F]FDG PET/MRI (**e**) and in DWI-sequences (**d**). In T1 fs Vibe the lesions are hard to detect (**c**). No signs of malignancy were seen in CT and bone scintigraphy (**a**, **b**)

**Fig. 3:**
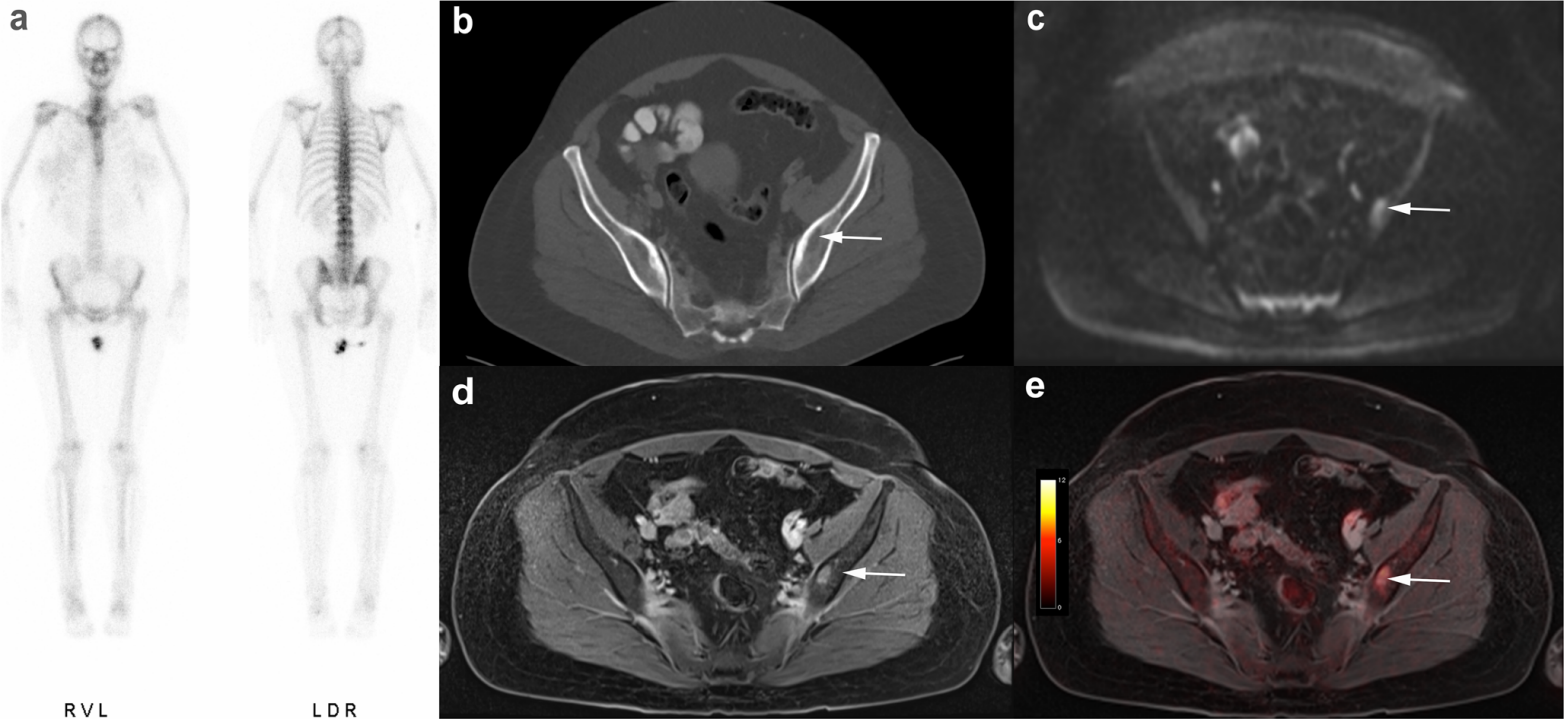
Forty-eight-year-old woman with breast cancer and a single histologically confirmed osteolytic metastasis in the left iliac bone. In the absence of cortical destruction, CT and bone scintigraphy yielded false-negative results (**a**, **b**). Clear identification of metastasis in MRI alone (**c**, **d**) and in fused [^18^F]FDG PET/MRI (**e**)

**Fig. 4:**
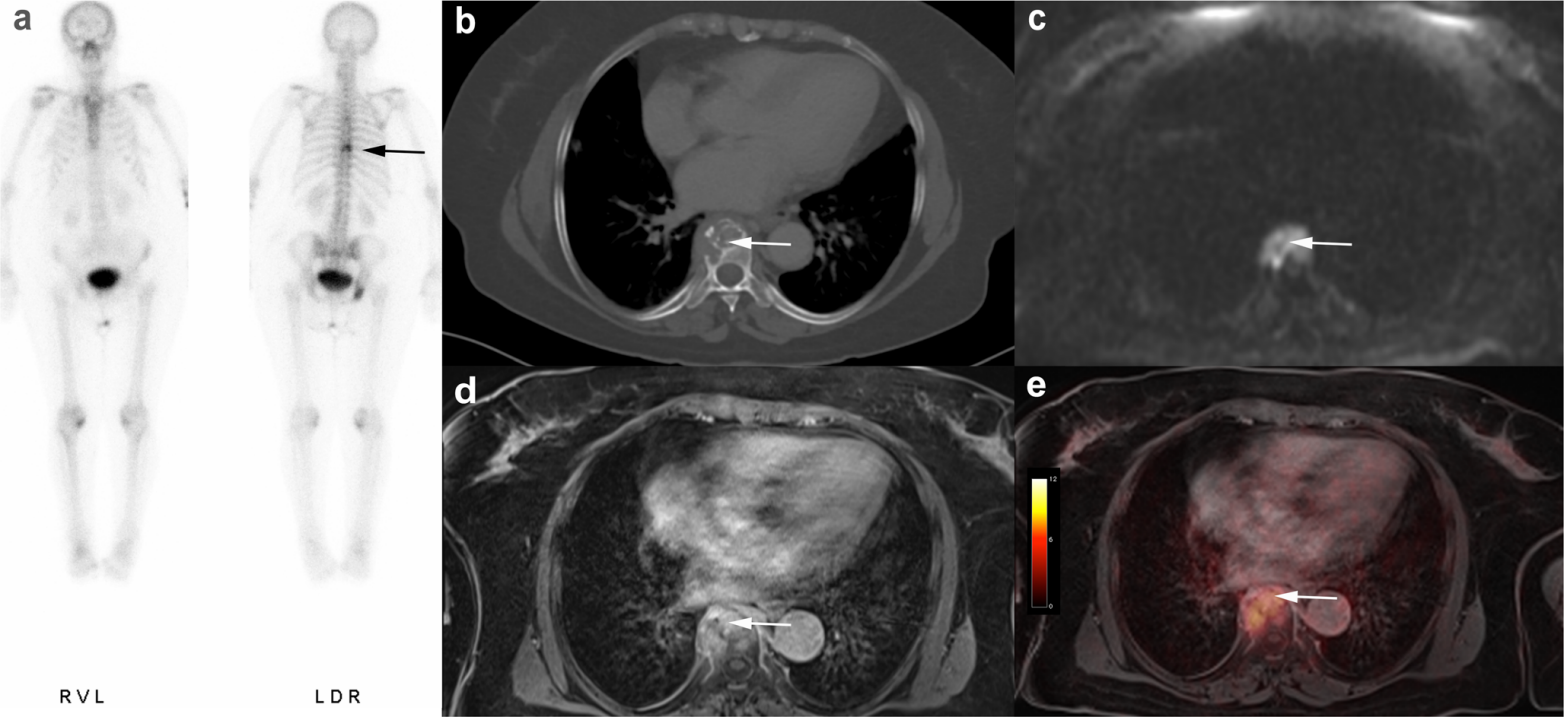
Seventy-five-year-old woman with breast cancer and histologically confirmed osteolytic bone infestation in thoracic vertebral body T8. All modalities show clear evidence of metastasis: focal accumulation in bone scintigraphy (**a**), cortical destruction and osteolysis in CT (**b**), diffusion restriction and contrast enhancement in MRI (**c**, **d**), and tracer uptake in fused [^18^F]FDG PET/MRI (**e**)

[^18^F]FDG PET/MRI did not miss any of the 41 malignant lesions (sensitivity 100%, CI: 79.0–100.0). All metastases showed a focal [^18^F]FDG uptake. There were no false-positive findings by [^18^F]FDG PET/MRI. MRI alone was also able to correctly identify all 41 metastases (sensitivity 100%, CI: 79.0–100.0), but misinterpreted one degenerative benign lesion as malignant. On MRI alone, the correct identification of 5 metastases was only possible through DWI, as they did not show a clear correlate on conventional morphologic MRI sequences (see Fig. 2). In comparison to that, CT detected 23 of 41 malignant bone lesions (sensitivity 56.1%, CI: 43.7–68.5; 8 lytic, 15 sclerotic). Fifteen malignant lesions were missed by CT and 3 sclerotic metastases were misinterpreted as bone islands. Especially osteolytic lesions of the bone marrow showing no signs of a tumor infestation such as cortical thinning or destruction were difficult to detect and often remained unrecognized on CT (Figs. 2, 3, and 4). Furthermore, there were 4 false-positive findings in CT, as 3 lesions in the axial skeleton and 1 lesion in a rib turned out to be degenerative or posttraumatic in histology and follow-up examination. Bone scintigraphy detected 15/41 bone metastases (sensitivity 36.6%, CI: 22.1–51.1; 7 lytic, 8 sclerotic) (Figs. 2, 3, and 4). Two of the lytic metastases detected with bone scintigraphy in one patient were not visible on CT. In addition, two posttraumatic lesions in the sternum and distal humerus in one patient were considered metastases in bone scintigraphy due to increased bone metabolism.

The McNemar *χ*^2^ test yielded statistical significance when comparing sensitivities in lesion detection of [^18^F]FDG PET/MRI with CT (*p* < 0.01, difference 43.9%, CI: 30.7–57.1) and MRI alone with CT (*p* < 0.01, difference 43.9%, CI: 30.7–57.1) as well as comparing [^18^F]FDG PET/MRI and MRI alone with bone scintigraphy (*p* < 0.01, difference 63.4%, CI: 40.7–76.1). The CT showed a statistically significant superiority in comparison to bone scintigraphy (*p* = 0.039, difference 19.5%, CI: 0.01–0.38).

### Diagnostic confidence

[^18^F]FDG PET/MRI showed a significantly higher overall diagnostic confidence in lesion nature ratings compared to MRI alone (4.16 ± 0.71 vs. 3.3 ± 0.65, *p* < 0.0001), CT (4.16 ± 0.71 vs. 3.04 ± 0.85, *p* < 0.0001), and bone scintigraphy (4.16 ± 0.71 vs. 3.93 ± 0.26, *p* = 0.0003). The difference of diagnostic confidence of MRI alone in comparison to CT did not reach statistical significance (3.3 ± 0.65 vs. 3.04 ± 0.85, *p* = 0.058).

## Discussion

In our study, [^18^F]FDG PET/MRI and MRI alone both outperform CT and bone scintigraphy when assessing bone metastases in the initial staging of patients with newly diagnosed breast cancer. On a lesion-based analysis, these modalities reveal a considerable advantage especially in the detection of osteolytic metastases. [^18^F]FDG PET/MRI shows no differences to MRI alone in sensitivity but offers a higher diagnostic confidence in correctly rating bone metastases. Bone scintigraphy achieved significantly worse results than CT in the detection of bone metastases.

Although the spectrum of available therapeutic options for breast cancer has significantly improved in recent years, distant metastases are still detectable in approximately one-third of patients during the course of the disease [3]. Bone metastases are by far the most frequent localization, accounting for 50–70% of distant metastases in the early phase of the disease [3–5]. Hence, a reliable initial staging has become increasingly important, as this allows for an individualized therapy regimen and early detection of patients with bone metastases to reduce skeletal morbidity by adjustment of chemotherapy, use of bisphosphonates, or targeted irradiation. Accordingly, the initial staging examination has recently been amended in guidelines, now including a thoraco-abdominal CT scan and a bone scintigraphy [12, 13]. Bone scintigraphy is still widely considered to be the gold standard in the detection of bone metastases, although a large number of studies in recent years have shown advantages of MRI as well as of hybrid imaging techniques [9, 15, 19, 20].

The results of our study raise the questions, whether a default bone scintigraphy is actually necessary in the primary staging of breast cancer patients when a CT is already performed and whether these two examinations should even remain the first choice considering the preeminent performance of PET/MRI or MRI alone. There have been various studies indicating that CT is superior to bone scintigraphy in detection of breast cancer metastases [21, 26, 27], and according to the results of a study by Bristow et al [27] comparing CT and bone scintigraphy in 44 patients with bone metastases from breast cancer, the routine bone scan may not be required. One advantage of our study is the relatively large, prospectively enrolled patient cohort undergoing initial staging based on current ESMO guidelines, hence reflecting clinical routine. Our results show that only 4% of patients have bone metastases in the initial breast cancer staging, which further questions the importance of bone scintigraphy in addition to thoraco-abdominal CT, also because breast cancer patients tend to be rather young and radiation dose should be considered. Nevertheless, in a clinical setting, review of both examinations is advisable in any case, especially since CT can facilitate the differentiation of benign and malignant radionuclide accumulations detected on bone scans [26].

Several studies have reported a superiority of whole-body MRI over CT and bone scintigraphy in bone lesions [15, 28], although it is rarely used for initial staging examinations of breast cancer in current clinical routine [16]. A major advantage of MRI is the possibility to directly visualize metastatic tissue in the bone marrow. Consequently, especially osteolytic metastases could be detected earlier than with CT and usually before cortical bone destruction has occurred [29, 30]. Furthermore, the early detection of a solitary bone metastasis might offer the opportunity of a curative approach by application of a local radiation therapy. According to our results, the visualization of osteolytic bone metastases with sole medullary involvement is highly limited both with CT and bone scintigraphy. Depending on the location, osteolytic metastases are detectable by bone scintigraphy only when approximately 50% of the bone marrow is already destroyed [21, 31]. This also has an influence on the therapy decisions. The earlier the metastases are discovered, the better they can be treated. As a result, this might prevent tumor-related osteolysis or fractures and reduce pain or other comorbidities [30]. MRI offers further advantages, such as the lack of ionizing radiation, or the higher soft tissue contrast, which might be beneficial for the detection of non-osseous lesions. In our study, diffusion-weighted MR imaging (DWI) revealed multiple metastases that would otherwise have been missed; hence, it should be considered part of the imaging protocol.

The ability of hybrid imaging to detect bone metastases in different tumor entities has been investigated extensively in recent years. PET/CT has already proven to be advantageous in cancer staging in comparison to CT and bone scintigraphy [20, 32–35].

The comparison of PET/CT and MRI has yielded conflicting results so far [36, 37]. In a study by Jambor et al with 26 high risk breast cancer patients, both modalities are described to be equally suitable for the detection of bone metastases with sensitivities of 93% and 91% [37]. The introduction of fully integrated PET/MRI in 2011 has enabled simultaneous acquisition of PET and high soft-tissue contrast morphological and functional MRI. In this study, the high sensitivity in detection of bone metastases is primarily caused by the [^18^F]FDG PET, but the combination with MRI offers the advantage of a high anatomical resolution and functional imaging. When comparing PET/CT and PET/MRI, available data is inconsistent. While Löfgren et al [38] did not see clear advantages of either one modality in the evaluation of bone metastases, studies of Sawicki et al [35] and Catalano et al [22] postulated a superiority of PET/MRI in recurrent breast cancer attributed to the higher soft tissue contrast and added information from functional imaging such as DWI.

Additionally to the mere detection of lesions, a high diagnostic confidence, allowing for a reliable differentiation between benign and malignant lesion nature, is relevant in daily routine. Although PET/MRI and MRI alone were able to detect all malignant bone lesions, our study emphasizes the level of diagnostic confidence achieved by hybrid imaging based on the ability to visualize pathologically increased glucose metabolism of malignant lesions [39].

This study has limitations. Despite the rather large study population with primary breast cancer, the number of patients with bone metastases was small. Second, in most cases, just one biopsy site has been chosen to histologically secure bone metastasis, since a histological sampling of all detected metastases is usually not required for determining the oncologic treatment concept. Therefore, the reference standard was also based on follow-up examinations using CT and MRI. Third, an adequate determination of a lesion-based specificity was not possible, since not all initially detected benign lesions were followed up with imaging. Fourth, up to 10% of osseous metastases in patients with breast cancer are located in the distal limbs and skull [40]. In this study, only lesions that could be detected by all modalities were included in the analysis. The thoracoabdominal CT had a slightly smaller FOV than the PET/MRI as the arms were raised above the head and were partly outside the FOV, while in PET/MRI the arms were lowered beside the body. So potential areas of metastasis may be excluded in this analysis, because of the limited FOV. Regardless, an additional separate evaluation of the complete FOV of each modality was performed but no additional metastases were found.

In conclusion, both [^18^F]FDG PET/MRI and MRI alone have shown to be significantly superior to CT and bone scintigraphy for the detection of bone metastases in patients with newly diagnosed breast cancer in our lesion-based analysis. MRI alone and [^18^F]FDG PET/MRI identified equivalent numbers of bone metastases. Considering the relatively low prevalence of bone metastases at initial diagnosis, the high number of patients at a relatively young age undergoing the clinical staging algorithm, and the therapeutic impact of bone metastases, radiation-free whole-body MRI might serve as modality of choice. In contrast, the use of currently recommended CT and bone scintigraphy for the detection of bone metastases seems questionable.

## Abbreviations

AC: Attenuation correction
ADC: Apparent diffusion coefficient
CI: Confidence interval
CT: Computed tomography
DWI: Diffusion-weighted imaging
EPI: Echo-planar imaging
ESMO: European Society For Medical Oncology
FDG: Fluorodeoxyglucose
FOV: Field of view
FWHM: Full-width at half maximum
HASTE: Half Fourier acquisition single shot turbo spin echo
HDP: Hydroxydiphosphonate
HER2: Human epidermal growth factor receptor 2
MRI: Magnetic resonance imaging
OSEM: Ordered-subset expectation maximization
PET: Positron emission tomography
RF: Radiofrequency
VIBE: Volume interpolated breath-hold examination
WHO: World Health Organization

## Declarations

### Guarantor

The scientific guarantor of this publication is Nils-Martin Bruckmann, MD.

### Statistics and biometry

Institute of Medical Informatics, Biometry and Epidemiology, University Hospital of Essen, Germany; Prof. Andreas Stang, head of the department.

### Methodology

• prospective

• diagnostic or prognostic study

• performed at two institutions
